# A New PCR-Based Method Shows That Blue Crabs (*Callinectes sapidus* (Rathbun)) Consume Winter Flounder (*Pseudopleuronectes americanus* (Walbaum))

**DOI:** 10.1371/journal.pone.0085101

**Published:** 2014-01-13

**Authors:** Jackie L. Collier, Sean P. Fitzgerald, Lyndie A. Hice, Michael G. Frisk, Anne E. McElroy

**Affiliations:** School of Marine and Atmospheric Sciences, Stony Brook University, Stony Brook, New York, United States of America; North Carolina State University, United States of America

## Abstract

Winter flounder (*Pseudopleuronectes americanus*) once supported robust commercial and recreational fisheries in the New York (USA) region, but since the 1990s populations have been in decline. Available data show that settlement of young-of-the-year winter flounder has not declined as sharply as adult abundance, suggesting that juveniles are experiencing higher mortality following settlement. The recent increase of blue crab (*Callinectes sapidus*) abundance in the New York region raises the possibility that new sources of predation may be contributing to juvenile winter flounder mortality. To investigate this possibility we developed and validated a method to specifically detect winter flounder mitochondrial control region DNA sequences in the gut contents of blue crabs. A survey of 55 crabs collected from Shinnecock Bay (along the south shore of Long Island, New York) in July, August, and September of 2011 showed that 12 of 42 blue crabs (28.6%) from which PCR-amplifiable DNA was recovered had consumed winter flounder in the wild, empirically supporting the trophic link between these species that has been widely speculated to exist. This technique overcomes difficulties with visual identification of the often unrecognizable gut contents of decapod crustaceans, and modifications of this approach offer valuable tools to more broadly address their feeding habits on a wide variety of species.

## Introduction

Winter flounder (*Pseudopleuronectes americanus*) historically supported robust commercial and recreational fisheries in the Long Island, New York region, with landings peaking in the early 1980s [1], [2]. However, by the 1990s winter flounder landings and population sizes began to decline, and since have fallen to just a few percent of their peak (http://www.st.nmfs.noaa.gov/) [1], [3], [4]. Winter flounder remain scarce in inshore and coastal waters and may be nearing extirpation in estuarine nurseries that once supported abundant populations [4], [5], [6], [7]. While harvest likely was a contributor to the decline of winter flounder, their failure to recover in the New York region despite a closure in Federal waters from 2009 to 2013 (http://www.nefmc.org) indicates additional factors may be involved. Surveys of juvenile winter flounder show that settlement of young-of-the-year (YOY) has not declined as sharply as adult abundance [1], [4], suggesting that post-settlement mortality of juveniles is a factor in the failure of winter flounder populations to recover.

Predation is thought to be a major cause of mortality in juvenile flatfish. Epibenthic predators including crangonid shrimp (such as the brown shrimp *Crangon crangon* and sand shrimp *Crangon septemspinosa* and *Crangon affinis*) and crabs (such as the green crab *Carcinus maenas*) have been shown to contribute significantly to the mortality of juvenile Japanese flounder (*Paralichthys olivaceus*) [8], [9], plaice (*Pleuronectes platessa*) [10] and winter flounder [11]. The smallest fish, particularly newly settled juveniles up to 20 mm in total length, are most vulnerable to crustacean predators, and juvenile flatfish do not reach a size refuge from crab predation until they exceed at least 50 mm in total length [9], [12], [13]. Additionally, unlike most flatfish, the adhesive, demersal eggs of winter flounder are also vulnerable to epibenthic predators. Taylor and Danila [14] have argued that warmer winters are contributing to the decline of winter flounder populations by increasing predation of *C. septemspinosa* on winter flounder eggs.

Recently, blue crab (*Callinectes sapidus*) landings have dramatically increased in the New York region to levels not observed since the 1870s [15], [16]. Blue crab landings have been shown to track abundance [17] and so it is likely the increase in harvest reflects population trends. Blue crab abundance may be responding to increasing temperature near the species' northern range limit, although the link between climate and blue crab abundance and distribution is complex [18], [19], [20]. To our knowledge, direct predation by blue crabs on juvenile winter flounder has yet to be demonstrated. The coincidence between the decline of winter flounder and increase of blue crabs in the New York region highlights the need to explore the trophic relationship between these species.

The diet of blue crabs varies with prey availability and has been reported to consist mainly of bivalve mollusks and crustaceans, including other crabs (e.g., [21], [22], [23], [24], [25], [26]), though fish can constitute a major portion of their diet [27]. These analyses have been based on visual examination of crab gut contents. However, some components of the diets of crabs and other decapod crustaceans are difficult to identify visually because crushing and maceration by the chelae and mouthparts during ingestion, combined with action of the gastric mill after ingestion, rapidly reduces soft parts of prey to unrecognizable material [11], [14], [23], [28]. Hard parts (such as otoliths or scales from fish) are more easily identified [29], [30] but are often not intact, making species-specific identification difficult, and otoliths and scales may be absent if the whole prey animal is not ingested [11].

Molecular genetic methods are increasingly being applied to overcome these kinds of obstacles in gut content analysis [31], [32]. In fact, the first use of species-specific polymerase chain reaction (PCR)-based methods to investigate predator-prey interactions was by Asahida et al. [33], who developed primers specific for the mitochondrial control region of stone flounder (*Kareius bicoloratus*) and found they could detect stone flounder DNA in the gut of laboratory-fed sand shrimp (*C. affinis*) for up to 5 hours at 9°C. Albaina et al. [10] used primers targeting the cytochrome b gene of plaice (*Pleuronectes platessa*) and a quantitative real-time PCR assay to monitor the digestion of plaice fed to brown shrimp (*C. crangon*) and shore crabs (*Carcinus maenas*) in the lab. Saitoh et al. [8] and Sudo et al. [9] used primers specific to the mitochondrial control region of Japanese flounder (*Paralichthys olivaceus*) to identify several species, including the crabs *Matuta lunaris*, *Portunus gladiator*, and *Charybdis japonica*, as predators of newly released hatchery-raised juvenile Japanese flounder. Here we report the development and validation of a method using winter flounder-specific oligonucleotide primers targeting the highly variable non-coding control region of the mitochondrial genome to specifically detect the presence of winter flounder in the natural diet of wild blue crabs.

## Materials and Methods

### Ethics statement

Collection and handling of experimental animals was conducted in accordance with permits issued to M.G. Frisk by the New York State Department of Environmental Conservation (#1030 and #1644), and the protocol for this study was approved by Stony Brook University’s Institutional Animal Care and Use Committee (IACUC; IRBNet#:260837-3, to A.E. McElroy).

### Primer design and PCR optimization

Sequences most similar to the *Pseudopleuronectes americanus* mitochondrial control region, GenBank accession number U12068.1, were identified by BLAST against the GenBank database (July 2011; http://www.ncbi.nlm.nih.gov/) and by additional GenBank database searches, downloaded, and aligned manually in BioEdit version 7 [34]. Four regions where the winter flounder sequence was unique were identified, each having at least 1 difference (typically many more) from all other flatfish and at least 2 differences (typically many more) from flatfish with geographical ranges overlapping winter flounder (Table S1). A forward primer was designed to match one region, and reverse primers were designed to match the other three regions, providing three possible pairs of winter flounder-specific primers expected to amplify PCR products between 92 and 208 bp in length (Table 1, WF primers). The WF primer pairs were nested within the region amplified by a second set of primers (one forward and two reverse), modified from primers developed by Lee et al. [35], and designed to amplify part of the control region from any flatfish species (Table 1, FF primers). Two different ‘universal’ primer pairs were used as positive controls for the presence of amplifiable DNA: one targeting an approximately 618 bp region of the 18S rRNA gene (Table 1, 18S primers from [36]), and another targeting a 176 bp region of the mitochondrial cytochrome c oxidase subunit I (COI) gene (*coxI*; Table 1, Uni-Minibar primers from [37]).

**Table 1:** Primers used in this study.

Name	Direction/position	Sequence 5’ to 3’	T_m_ °C	GC%	Product length in base pairs (with forward primer)	Primer pair name
WF200f	For, 75–93	ATAATGAACTAGGACATCT	44.4	31.5	--	--
WF270r	Rev, 150–167	AATAGGTTTCAGTAAATC	40.8	27.7	92 (with WF200f)	WF92
WF310r	Rev, 185–202	GTCCTGGACTTTCAGATG	49.8	50.0	127 (with WF200f)	WF127
WF400r	Rev, 266–283	ATACGAATTTGAGTTGGA	45.5	33.3	208 (with WF200f)	WF208
FF_A	For, <1	CCCTAACTCCCAAAGCTAG	52.0	52.6	--	--
FF_2	Rev, 382–401	CCTGAAGTAGGAACCAAATG	50.7	45.0	439–459 (with FF_A)^a^	FF450
FF_3	Rev, 465–482	TGGGTAACGAGTCGTATG	51.1	50.0	519–540 (with FF_A)^a^	FF530
18S-A	For, <1–19	AACCTGGTTGATCCTGCCAGT	58.9	52.4	--	--
18S-570R	Rev, 597–616	GCTATTGGAGCTGGAATTAC	58.8	45.0	618 (with 18S_A)^a^	18S
Uni-MinibarF1	For, 3–28	TCCACTAATCACAARGATATTGGTAC	53.5	36.5	--	--
Uni-MinibarR1	Rev, 156–179	GAAAATCATAATGAAGGCATGAGC	52.8	37.5	176 (with Uni-MinibarF1)	Unibar

For: forward primer. Rev: reverse primer. For mitochondrial control region primers (WF and FF), position in winter flounder sequence U12068 is given; for 18S primers, position in EU637075, *Kareius bicoloratus* 18S rRNA gene is given; for Uni-Minibar primers, position in HM180652, *Pseudopleuronectes yokohamae* cytochrome oxidase subunit I (COXI) is given. T_m_: melting temperature predicted by Integrated DNA Technologies’ Oligoanalyzer program (http://www.idtdna.com/analyzer/applications/oligoanalyzer/). GC%: % GC content of primer.

^a^ exact length varies between species.

Initial testing and optimization of PCR conditions for WF and FF primer pairs were performed using genomic DNA extracted from winter flounder, summer flounder (an abundant co-occurring flatfish at our field site), and blue crab muscle tissue (from animals collected concurrently with our field study) using a QIAGEN DNeasy Blood and Tissue Kit (QIAGEN, Valencia, CA, USA) and following the spin-column protocol for animal tissue. Primers were synthesized by Integrated DNA Technologies (IDT, Coralville, IA, USA), and were diluted to a 5 µM working stock. Each 20 µL PCR reaction contained: 2 µL BSA (3 mg/mL, New England BioLabs, Ipswich, MA, USA), 2 µL dNTPs (2 mM), 2 µL MgCl_2_ (25 mM), 2 µL Mg Free buffer (10X), 2 µL of each primer (5 µM), 0.2 µL GoTaq Flexi Polymerase (5 u/µL), 6.8 µL distilled water, and 1 µL of DNA template. All reagents were purchased from Promega (Madison, WI, USA) unless otherwise specified. Neither the WF92 nor the WF127 primer pair amplified the expected product from winter flounder DNA, even at low annealing temperatures, and so these primers were excluded from further consideration. Annealing temperature was optimized using a Techne (Bibby Scientific Limited, Staffordshire, UK) Touchgene Gradient thermocycler, and routine PCR reactions with WF208, FF450 and FF530 primer pairs were carried out using a 2 minute initial denaturation at 95°C followed by 35 cycles of 30 seconds at 95°C, 30 seconds at 50°C, and 90 seconds at 72°C, with a final extension for 10 minutes at 72°C and hold at 4°C. The same PCR conditions were used for the 18S primer pair, while reactions with the Unibar primer pair were done following the thermal cycling protocol from Meusnier et al. [37]. Amplicons were visualized by electrophoresis in 2% agarose gels with 1X Tris-Acetate-EDTA (TAE) buffer and staining with ethidium bromide.

### Blue crab gut contents analysis method development

Blue crabs were collected using a 1 m beam trawl deployed for 5 minutes in Shinnecock Bay (on the south shore of Long Island, NY, USA) during the summer of 2011. Crabs collected for gut content analysis were routinely placed on wet ice in the field and transferred to storage in a -80°C freezer upon return to the lab, although some crabs were instead frozen on dry ice in the field immediately after collection to compare preservation techniques. Crabs used in feeding experiments were kept damp on ice in a cooler and transferred to seawater in the laboratory.

Foregut contents were removed from thawed crabs using instruments rinsed in ethanol and flame sterilized in an alcohol lamp. Liquid and solid gut contents were combined in a 1.5 mL microcentrifuge tube and either assayed immediately or frozen at –80°C until further analysis. Two different methods of homogenization were tested on gut contents of wild crabs diluted with 4 volumes of 1X TE (10 mM Tris-HCl, 1.0 mM EDTA, pH 7.5): a disposable pellet pestle operated for 30 seconds by hand then for 30 seconds with a battery-operated homogenizer (Kontes Pellet Pestle Motor, Kimball Chase, Vineland, NJ, USA), or glass beads (mixed sizes from 0.15 to 4 mm in diameter) using a Mini-Beadbeater for 60 seconds (BioSpec Products, Bartlesville, OK, USA). To create positive controls, some subsamples were supplemented with 0.25 mg of winter flounder tissue before homogenization to simulate the presence of winter flounder tissue in natural blue crab gut contents. DNA was extracted from 100 µL aliquots of homogenized gut material as described above for muscle tissues. All extracted DNA was stored at –20°C. Amplification with WF208, FF530, and 18S primer pairs showed no difference between the pestle and bead-beating homogenization methods, and also no difference between crabs frozen immediately on dry ice in the field and those held on wet ice until transfer to storage at –80°C (data not shown). For practical reasons, all further experiments were performed using crabs held on wet ice in the field and using homogenization by pestle.

Preliminary experiments showed that lengthy processing time, either during crab thawing and dissection or after gut contents removal, diminished recovery of amplifiable DNA (data not shown). DNA degradation during dissection and gut contents processing was minimized by removing one crab at a time from the freezer and placing it under a running stream of warm water until thawed just enough (1 to 2 minutes) to allow dissection of the still partially-frozen crab. Effort was also made to minimize inclusion of blue crab tissue in the gut contents samples. 1/5 volume of 0.5X TE (5 mM Tris-HCl, 0.5 mM EDTA, pH 7.5) was immediately added to collected gut contents, and the sample was kept on ice (not refrozen) until a few samples were ready for further processing (within one hour of dissecting the first crab).

Blue crabs returned to the lab alive were starved for 24 hours and then used in a feeding experiment designed to determine how long winter flounder DNA remained detectable during normal digestion in live crabs kept at 23°C. Crabs were frozen at –80°C from 1 to 24 hours after feeding on a piece of adult winter flounder muscle tissue (1 to 2 g), with crabs that did not consume winter flounder tissue being frozen as negative controls. Gut contents were collected and homogenized as described above, DNA was extracted from a 30 µL subsample, and subjected to amplification with the WF208 and either Unibar or 18S primer pairs.

### Detection of winter flounder DNA in wild blue crabs

Blue crabs were collected from three sites in Shinnecock Bay during summer and fall of 2011 using 3–5 minute tows of a 5 m otter trawl as part of a larger study of the macrofauna at this location (K. Rountos, unpublished data). Macroinvertebrates and fish were separated into different bins, and up to five blue crabs per trawl were placed on ice no more than forty minutes after they were captured, transported back to the lab on ice and frozen at –20°C. Juvenile winter flounder were also caught at these sites, though not always in the same trawls as blue crabs. Fifty-five blue crabs (carapace width >54 mm) were selected for gut contents analysis (dates and locations of capture along with other characteristics of each crab are shown in Table S2) by the method described for the feeding experiment.

### DNA Sequencing

Selected PCR products were treated with ExoSAP-It in accordance with the manufacturer’s protocol (Affymetrix, Santa Clara, CA, USA), combined with an appropriate primer and submitted to the Stony Brook University DNA Sequencing Facility. The resulting sequences were identified by comparison against GenBank using BLAST. Sequences long enough to be submitted are available in GenBank under Accession Numbers KF183644-KF183646 and shorter sequences are shown in Figures S1 and S2.

## Results

**Figure 1:**
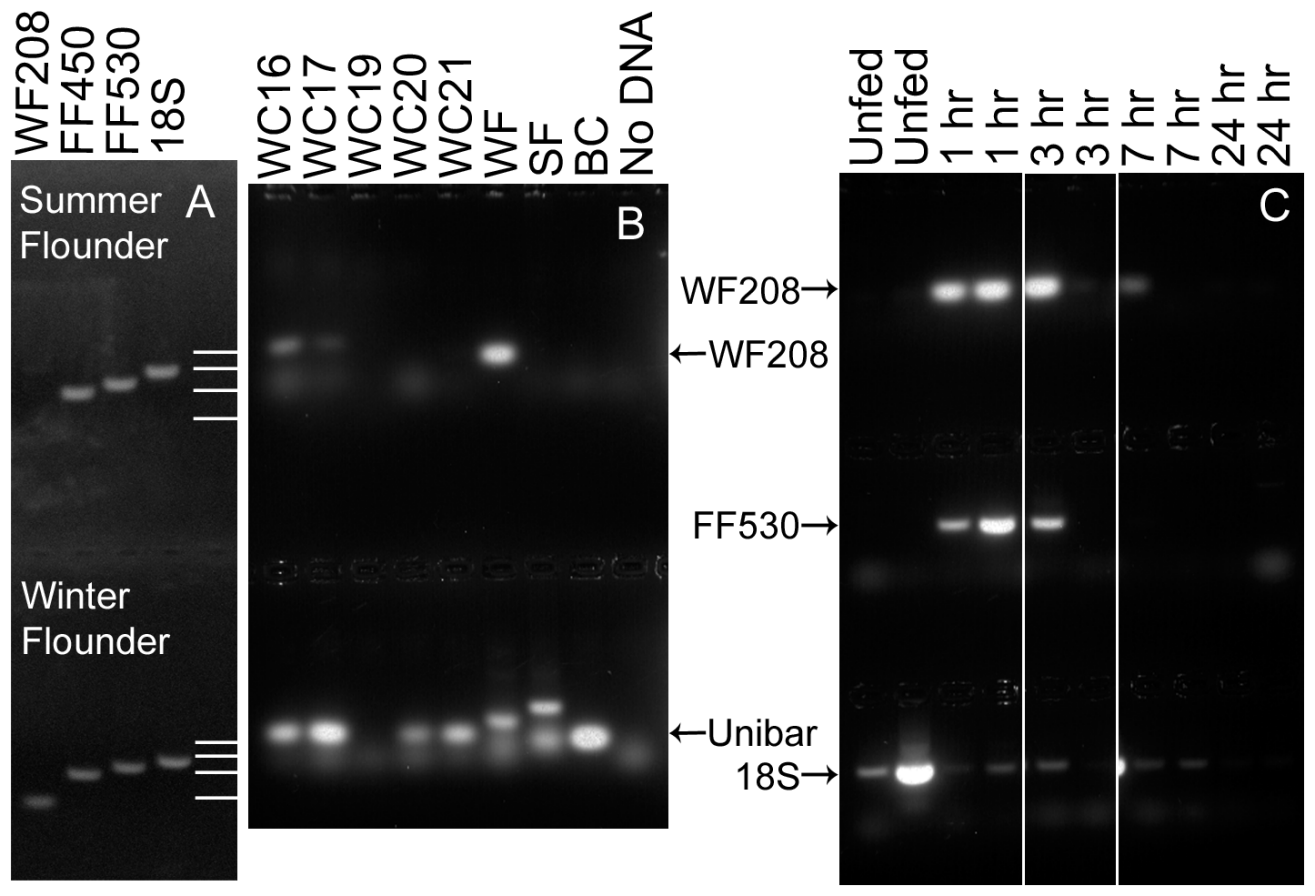
Selected PCR results. (1a) PCR amplification of DNA purified from summer flounder (top) and winter flounder (bottom) by primer pairs WF208, FF450, FF530, and 18S. Positions of DNA size standards (1000, 750, 500, and 250 base pairs) indicated in the right column. (1b) PCR amplification of DNA purified from five wild crabs (WC16, WC17, WC19, WC20, WC21), winter flounder (WF), summer flounder (SF), and blue crab (BC) by primer pairs WF208 (top) and Unibar (bottom); last lane shows no DNA negative controls. WC16 and WC17 were positive for both WF208 and Unibar, WC19 was negative for both, while WC20 and WC21 were negative for WF208 and positive for Unibar. (1c) PCR amplification using primer pairs WF208 (top), FF530 (middle) and 18S (bottom) of DNA purified from the gut contents of 12 crabs from the feeding experiment. The first two lanes show unfed, negative control crabs, and the remaining lanes show crabs frozen at various times (1, 3, 7 or 24 hours) after feeding on winter flounder tissue. There was a faint WF208 band in the second 3 hour crab, a faint FF530 band in the first 7hr crab, and 18S bands were present in all samples.

The WF208 primer pair amplified a product of the expected size from winter flounder DNA, and did not amplify anything from summer flounder or blue crab DNA, whereas both general flatfish primer pairs FF450 and FF530 amplified products of the expected size from both winter and summer flounder DNA (Figures 1a, 1b) but nothing from blue crab DNA (data not shown). The 18S primer pair amplified products of the expected size from winter flounder and summer flounder DNA (Figure 1a) as well as blue crab DNA (data not shown). In contrast, the Unibar primer pair did not reliably amplify products of the expected size from either winter or summer flounder DNA (sometimes producing nothing, multiple bands, or larger-than-expected products) but did always amplify a single product of the expected size from blue crab DNA (Figure 1b), and was routinely used as a positive control for extraction of amplifiable DNA from crab gut contents. The amount of primer-dimer produced by the WF208 and Unibar primers varied among samples (Figure 1).

The feeding experiment demonstrated that the WF208 and FF530 primer pairs were able to amplify WF DNA from the gut contents of crabs frozen from 1 to 7 hours after consuming winter flounder tissue, but not from crabs frozen 24 hours after consuming winter flounder tissue or crabs that did not consume winter flounder while captive in the lab (Figure 1c). Sequencing of PCR products from these experiments confirmed that the WF208 and FF530 primer pairs both amplified the expected regions of winter flounder DNA (Figure S1and GenBank Accession number KF183646, respectively). The 18S product from some samples was identical to the 18S rRNA sequences of several other flatfish (KF183644 versus *Hippoglossoides dubius*, AB112469; *Cleisthenes herzensteini*, EF126039; *Zebrias zebra*, EF126044; and *Kareius bicoloratus*, EU637055), representing winter flounder, while from other samples it was either identical to the 18S sequence of blue crab (KF183645 versus AY781436) or the PCR product was a mixture of two (or more) different sequences (data not shown).

**Figure 2:**
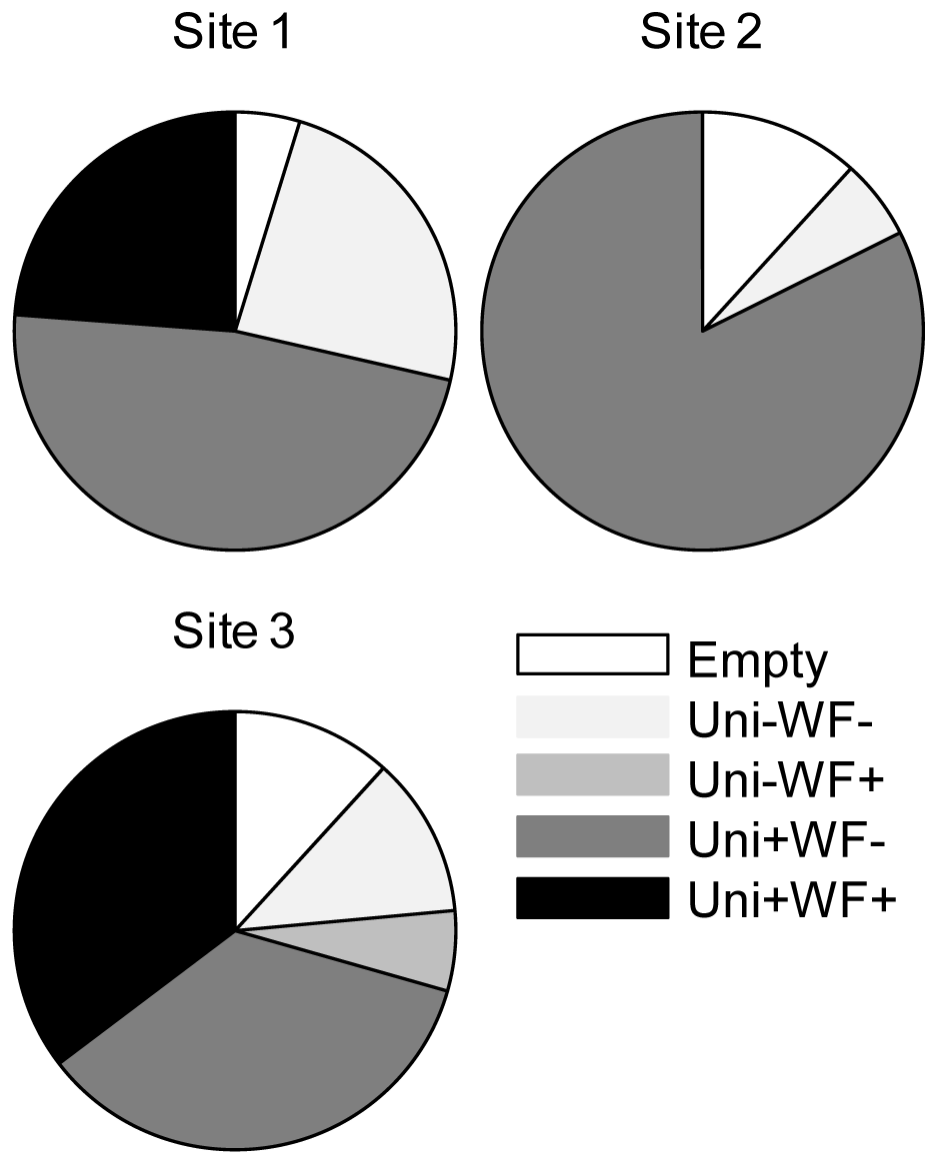
Detection of winter flounder DNA in the gut contents of 55 wild crabs. Categories of crab gut contents found at site 1 (n = 21), site 2 (n = 17), and site 3 (n = 17): Empty, crabs with empty foreguts; Uni-WF-, no amplification with either Unibar or WF208 primers; Uni-WF+, no amplification with Unibar primers but successful amplification with WF208 primers; Uni+WF-, successful amplification with Unibar primers but no amplification with WF208 primers; Uni+WF+, successful amplification with both Unibar and WF208 primers.

Detection of amplifiable winter flounder DNA in wild crab gut contents varied widely between sites, and by date of collection. Of the 55 wild crabs analyzed, 5 had empty guts (Figure 2, Table S2). Of the remaining 50, 41 yielded PCR products of the expected size with the Unibar primers. Of those 41, 11 also yielded PCR products of the expected size with the WF208 primers. One crab (WC46) also yielded the WF208 product even though it did not yield a Unibar product. Overall, the diets of 12 of 55 blue crabs (21.8%) included winter flounder. Excluding crabs with empty guts or from which no amplifiable DNA was recovered raises the proportion to 28.6% (12 of 42). At one site no crabs tested positive for winter flounder DNA, while the proportion of winter flounder-positive crabs at the other two sites (excluding empty and unamplifiable samples) was 33.3% and 53.9%. The proportion of WF208-positive crabs at each site varied greatly by date, from 0 to 83.3%. Blue crabs from site 1 yielded amplifiable winter flounder DNA only on the first of six dates (August 4) while blue crabs from site 3 yielded amplifiable winter flounder DNA on three of six dates (August 10, 18, and 30). Sequencing of four WF208 PCR products confirmed the specific amplification of the targeted winter flounder mitochondrial control region from wild blue crab gut contents (Figure S1). Sequences of Unibar PCR products from 11 wild crabs were either identical to the blue crab *coxI* sequence (NC_006281; Figure S2) or similar to the blue crab *coxI* but mixed with other sequences (data not shown).

## Discussion

This work presents the first empirical evidence that wild blue crabs are consuming wild winter flounder. Our overall values, in the range of 20 to 30% WF208-positive crabs, are similar to the prevalence (14% and 33% in two years of study) of Japanese flounder (*Paralichthys olivaceus*) DNA found in Asian paddle crab (*Charybdis japonica*) in the days following a large release of vulnerable hatchery-reared juvenile flounder [9]. Sudo et al. [9] found no *P. olivaceus* DNA in *C. japonica* before the hatchery flounder were released, so it is not clear whether *C. japonica* consume wild *P. olivaceus*. Our overall values are also similar to the 18% prevalence of “finfish” in blue crab gut contents reported by Ropes [23] but lower than the 60% reported by Fitz and Wiegert [27]. Studies of *Crangon* spp. reported 7.2% of sand shrimp tested positive for winter flounder [14] and 5% of bay shrimp positive for plaice [13]. The consistency and amount of gut contents present varied among crabs, even those fed in the lab and sacrificed at the same time, and did not differ systematically between blue crabs testing positive or negative for winter flounder, except that no gut containing only light colored liquid or less than 100 µL volume (typical for unfed crabs in the lab feeding experiments) tested positive for winter flounder DNA. Laughlin [26] found that all blue crabs with carapace width greater than 60 mm had similar feeding habits, and we did not detect any differences in sex or size between crabs that were positive or negative for winter flounder DNA.

Several technical issues may have caused us to underestimate the prevalence of winter flounder in blue crab gut contents. One potential cause of false negative results (failure to detect winter flounder DNA in a crab that had recently consumed winter flounder) is the primers being unable to amplify all variants of the winter flounder mitochondrial control region. Having only one winter flounder mitochondrial control region sequence available when these primers were designed increased the risk that sequencing errors or biological variation could cause this problem. The existence of biological variation was confirmed by the finding that our FF530 sequence (KF183646) differed from U12068 at 14 of 464 positions, and the WF208 sequences differed from U12068 at an additional 4 (Figure S1). This variation included one mismatch to the WF200f primer, up to three mismatches to the WF270r primer, up to two mismatches to the WF310r primer, and one mismatch to the WF400r primer. The mismatches in the WF270r and WF310r primer sequences could explain the poor performance of the WF92 and WF127 primer pairs, and suggest the need for a sequencing effort to define the natural variation in the winter flounder mitochondrial control region.

Another potential cause of false negative results is inhibition of the PCR reaction by compounds co-purified with the template DNA, as reported by Albaina et al. [10] for gut contents of the crab *Charybdis japonica*. However, our tests of PCR inhibition, performed by adding a small amount of winter flounder DNA to PCR reactions containing template DNA that did not amplify with WF208 or 18S primers, showed no evidence of inhibition (data not shown). Instead, either the targeted regions were not present or the DNA in these samples was too degraded to support amplification. Template DNA may become too degraded due to technical issues, such as failure to process gut contents quickly and with adequate safeguards. Our method for processing the wild crabs minimized these concerns by limiting the time between crab dissection and DNA purification from a subsample of its gut contents to at most 1 hour with no freeze/thaw cycles. We used the more universal 18S and Unibar primer pairs to confirm the presence of amplifiable DNA in each sample, with the Unibar primers preferred because the product was of similar size to the WF208 product, and even though the Unibar primer pair did not reliably amplify winter flounder DNA, blue crab DNA was expected to be present in every sample.

Our study may also have underestimated the prevalence of winter flounder in blue crab gut contents if crabs were captured too long after they had consumed winter flounder. Our controlled lab feeding experiments showed that our method was able to detect winter flounder DNA in the gut contents of blue crabs for up to 7 hours, but not for 24 hours, at 23°C. This result is consistent with previous reports on the gut passage time of blue crabs, which were found to empty their stomach between 8 and 10 hours after feeding at 20°C [38]. We expect that digestion occurred at a similar rate in our wild Shinnecock Bay crabs, which were collected when the water temperature was ∼24°C (L.A. Hice, unpublished data). Reports on the time of day when blue crabs feed most actively vary, with some studies reporting crepuscular or nocturnal feeding [24], [30], while others reported more active daytime feeding [27] or no difference with time of day [26]. A better understanding of both the timing of blue crab feeding and gut passage time will be required to draw quantitative conclusions from studies of gut contents.

Another factor that may have limited our detection of winter flounder DNA in blue crabs is the seasonal timing of our study. We focused on relatively large (>50 mm carapace width) crabs for ease of collecting gut contents. Crabs of this size were collected primarily in late July and August. In contrast, in Shinnecock Bay and at many other shallow nearshore sites around Long Island, abundance of juvenile winter flounder peaks in late June [4] (L.A. Hice, unpublished data), and consumption of winter flounder by blue crabs may be more common when flounder are more abundant.

The presence of winter flounder DNA in blue crab gut contents may indicate predation by one blue crab on one live winter flounder, or may instead reflect a number of other possible biological interactions, including scavenging of moribund or dead fish, consumption of one flounder by multiple crabs, secondary predation (the crab having eaten a prey item that had first eaten winter flounder), and predation occurring in the net during sampling. Although Paul [30] argued that portunid crabs are unlikely to be effective hunters of finfish, a variety of later studies have reported predation by portunid crabs on finfish, particularly juvenile flatfish, both in the lab and in the field [8], [9], [12], [13], [22], [23], [24], [26], [27], [28], [39]. The lower contribution of flatfish to the diets of *Crangon* spp. than portunid crabs [9], [13], [14], [23], [27] could reflect the vulnerability of only the very smallest fish to shrimp; for example, winter flounder reach a size refuge from 7-spine bay shrimp at approximately 20 mm [40]. Juvenile winter flounder collected in Shinneocock Bay in August 2011 as part of a larger study investigating winter flounder in Long Island bays ranged from 51 to 121 mm total length, with a mean of 82.9 mm (L.A. Hice, unpublished data). Based on a study of green crab predation [12], all of the blue crabs examined in this study were likely large enough (>50 mm carapace width) to prey on juvenile winter flounder up to at least 50 mm total length and likely larger. In regard to secondary predation, other crabs and sand shrimp are the most likely organisms to both prey upon winter flounder and become prey for blue crabs; the frequency of such interactions and length of time for which secondarily ingested winter flounder DNA would be detectable in blue crab gut contents are unknown, but this seems a less likely explanation for our results than direct predation [41]. It is unlikely that our results represent false positives caused by predation in the net during collection because no winter flounder were caught in at least one of the trawls that yielded many WF208-positive blue crabs (August 4, 2011 at site 1). Additionally, no crabs were ever seen eating anything while in the trawl nets, and it is unlikely such behavior would have gone unnoticed. Further studies will be required to evaluate the potential roles of scavenging and kleptoparasitism in consumption of winter flounder by blue crabs.

A review of historical data suggests that the abundance of blue crabs in the Long Island region has varied substantially over the last two centuries [16], [42]. The peak in reported landings occurred in the 1880s and underwent a slow decline until the fishery was nearly absent by the 1930s [15]. More recently, blue crab landings have increased and are approaching the peak levels of the 1880s [42]. Ecosystem models of Great South Bay (nearby Shinnecock Bay on the south shore of Long Island) show that high blue crab abundance during the 19th century corresponded to a period of low winter flounder abundance [16], [42]. Although our study does not provide direct evidence that blue crab predation can control winter flounder populations, it does demonstrate a potential mechanism for a negative correlation between these two species. If direct predation is the process most responsible for the presence of winter flounder DNA in blue crab gut contents, this and previous studies indicate that blue (and other portunid) crabs could impose substantial mortality on juvenile winter flounder on the south shore of Long Island and in other places where these species co-exist. This study cannot (and was not designed to) provide a quantitative assessment of blue crab predation on winter flounder. Since it appears critical for future management of the south shore bays of Long Island that the predator and prey dynamics of blue crabs and winter flounder be elucidated, particularly in response to the current increase in blue crab abundance, further work should be done to survey crabs during the entire spring and summer seasons, from winter flounder egg deposition through growth of juveniles into a size refuge, to produce a quantitative estimate of this trophic interaction.

## Supporting Information

Figure S1 ***Pseudopleuronectes americanus*** **mitochondrial D-loop sequences.** The alignment starts from position 1 of GenBank Accession Number U12068, and compares U12068 to sequences recovered with the WF208 primer pair from wild blue crab gut contents spiked with winter flounder tissue (HB-WF208), from feeding experiment crabs (GenBank Accession KF183646, FC2-WF208, FC4-WF208), and from wild crabs (WC14-WF208, WC15-WF208, WC40-WF208, and WC46-WF208). The locations and sequences of primers described in Table 1 are also shown.
(PDF)

Figure S2 ***Calinectes sapidus coxI*** **sequences.** The alignment starts from position 1276 of GenBank Accession Number NC_006281 and compares NC_006281 to the consensus of sequence recovered from wild blue crabs by the Unibar primer pair. The locations and sequences of primers described in Table 1 are also shown.
(PDF)

Table S1 Number of sequence differences (mismatches plus insertion/deletions) between mitochondrial control region primers listed in Table 1 and control region sequences from flatfish and blue crab.
(PDF)

Table S2 Characteristics and amplification results of the 55 crabs from Shinnecock Bay used in wild crab analysis.
(PDF)

## References

[1] Socrates J, Colvin G (2006) A study of the striped bass in the Marine District of New York State. Completion Report for Project AFC-30. New York State Department of Environmental Conservation, East Setauket, NY.

[2] Vonderweidt C (2006) Species Profile: Winter Flounder current plan seeks to Rebuild Southern New England/Mid-Atlantic Stock and sustain Gulf of Maine Stock. ASMFC Fisheries Focus 15: 4–6.

[3] NEFSC (2011) 51st Northeast regional stock assessment workshop (51st SAW) assessment report. NEFSC Ref. Doc. 11-02. Northeast Fisheries Science Center. 856 p.

[4] Yencho MA (2009) Abundance, mortality, age and growth of young-of-the-year winter flounder (*Pseudopleuronectes americanus*) in two locations on Long Island. Stony BrookNY: Stony Brook University. 91 p.

[5] CT DEEP (2011) A study of marine recreational fisheries in Connecticut. Federal Aid in Sport Fish Restoration F54-R-31 Annual Performance Report. Department of Energy and Environmental Protection. 275 p.

[6] DeLong AK, Collie JS, Meise CJ, Powell JC (2001) Estimating growth and mortality of juvenile winter flounder, *Pseudopleuronectes americanus*, with a length-based model. Canadian Journal of Fisheries and Aquatic Sciences 58: 2233–2246.

[7] O’Leary SJ, Hice LA, Feldheim KA, Frisk MG, McElroy AE, et al (2013) Severe inbreeding and small effective number of breeders in a formerly abundant marine fish. PLoS ONE 8: e66126. 23762473 10.1371/journal.pone.0066126 PMC3676343

[8] Saitoh K, Takagaki M, Yamashita Y (2003) Detection of Japanese flounder-specific DNA from gut contents of potential predators in the field. Fisheries Science 69: 473–477.

[9] Sudo H, Kajihara N, Fujii T (2008) Predation by the swimming crab *Charybdis japonica* and piscivorous fishes: A major mortality factor in hatchery-reared juvenile Japanese flounder *Paralichthys olivaceus* released in Mano Bay, Sado Island, Japan. Fisheries Research 89: 49–56.

[10] Albaina A, Fox CJ, Taylor N, Hunter E, Maillard M, et al (2010) A TaqMan real-time PCR based assay targeting plaice (*Pleuronectes platessa* L.) DNA to detect predation by the brown shrimp (*Crangon crangon* L.) and the shore crab (*Carcinus maenas* L.)-Assay development and validation. Journal of Experimental Marine Biology and Ecology 391: 178–189.

[11] Taylor DL (2004) Immunological detection of winter flounder (*Pseudopleuronectes americanus*) eggs and juveniles in the stomach contents of crustacean predators. Journal of Experimental Marine Biology and Ecology 301: 55– 73.

[12] Fairchild EA, Howell WH (2000) Predator-prey size relationship between *Pseudopleuronectes americanus* and *Carcinus maenas* . Journal of Sea Research 44: 81–90.

[13] van der Veer HW, Bergman MJN (1987) Predation by crustaceans on a newly settled 0-group plaice *Pleuronectes platessa* population in the western Wadden Sea. Marine Ecology Progress Series 35: 203–215.

[14] Taylor DL, Danila DJ (2005) Predation on winter flounder (*Pseudopleuronectes americanus*) eggs by the sand shrimp (*Crangon septemspinosa*). Canadian Journal of Fisheries and Aquatic Sciences 62: 1611–1625.

[15] Briggs PT (1998) New York's blue crab (*Callinectes sapidus*) fisheries through the years. Journal of Shellfish Research 17: 487–491.

[16] Nuttall MA, Jordaan A, Cerrato RM, Frisk MG (2011) Identifying 120 years of decline in ecosystem structure and maturity of Great South Bay, New York using the Ecopath modelling approach. Ecological Modeling 222: 3335–3345.

[17] Colton AR (2011) An evaluation of the synchronization in the dynamics of blue crab (*Callinectes sapidus*) populations in the western Atlantic: University of Maryland, College Park. 247 p.

[18] Bauer L, Miller T (2010) Spatial and interannual variability in winter mortality of the blue crab (*Callinectes sapidus*) in the Chesapeake Bay. Estuaries and Coasts 33: 678–687.

[19] Bauer LJ, Miller TJ (2010) Temperature-, salinity-, and size-dependent winter mortality of juvenile blue crabs (*Callinectes sapidus*). Estuaries and Coasts 33: 668–677.

[20] Hines AH, Johnson EG, Darnell MZ, Rittschof D, Miller TJ, et al. (2010) Predicting effects of climate change on blue crabs in Chesapeake Bay. In: Kruse GH, Eckert GL, Foy RJ, Lipcius RN, Sainte-Marie B et al.., editors. Biology and Management of Exploited Crab Populations under Climate Change. Fairbanks: Alaska Sea Grant, University of Alaska Fairbanks. pp. 109–127.

[21] Hines AH, Haddon AM, Wiechert LA (1990) Guild structure and foraging impact of blue crabs and epibenthic fish in a subestuary of Chesapeake Bay. Marine Ecology Progress Series 67: 105–126.

[22] Meise CJ, Stehlik LL (2003) Habitat use, temporal abundance variability, and diet of blue crabs from a New Jersey estuarine system. Estuaries 26: 731–745.

[23] Ropes JW (1989) The food habits of five crab species at Pettaquamscutt River, Rhode Island. Fishery Bulletin 87: 197–204.

[24] Ryer CH (1987) Temporal patterns of feeding by blue crabs (*Callinectes sapidus*) in a tidal-marsh creek and adjacent seagrass meadow in the lower Chesapeake Bay. Estuaries 10: 136–140.

[25] Stehlik LL, Pikanowski RA, McMillan DG (2004) The Hudson-Raritan Estuary as a crossroads for distribution of blue (*Callinectes sapidus*), lady (*Ovalipes ocellatus*), and Atlantic rock (*Cancer irroratus*) crabs. Fishery Bulletin 102: 693–710.

[26] Laughlin RA (1982) Feeding habits of the blue crab, *Callinectes sapidus* Rathbun, in the Apalachicola Estuary, Florida. Bulletin of Marine Science 32: 807–822.

[27] Fitz HC, Wiegert RG (1991) Utilization of the intertidal zone of a salt marsh by the blue crab *Callinectes sapidus*: density, return frequency, and feeding habits. Marine Ecology Progress Series 76: 249–260.

[28] Taylor DL (2005) Predatory impact of the green crab (*Carcinus maenas* Linnaeus) on post-settlement winter flounder (*Pseudopleuronectes americanus* Walbaum) as revealed by immunological dietary analysis. Journal of Experimental Marine Biology and Ecology 324: 112– 126.

[29] Wilcox JR, Jeffries HP (1974) Feeding habits of the sand shrimp *Crangon septemspinosa* . Biological Bulletin 146: 424–434. 10.2307/1540689 28368207

[30] Paul RKG (1981) Natural diet, feeding and predatory activity of the crabs *Callinectes arcuatus* and *C. toxotes* (Decapoda, Brachyura, Portunidae). Marine Ecology Progress Series 6: 91–99.

[31] King RA, Read DS, Traugott M, Symondson WOC (2008) Molecular analysis of predation: a review of best practice for DNA-based approaches. Molecular Ecology 17: 947–963. 18208490 10.1111/j.1365-294X.2007.03613.x

[32] Symondson WOC (2002) Molecular identification of prey in predator diets. Molecular Ecology 11: 627–641. 11972753 10.1046/j.1365-294x.2002.01471.x

[33] Asahida T, Yamashita Y, Kobayashi T (1997) Identification of consumed stone flounder, *Kareius bicoloratus* (Basilewsky), from the stomach contents of sand shrimp, *Crangon affinis* (De Haan) using mitochondrial DNA analysis. Journal of Experimental Marine Biology and Ecology 217: 153–163.

[34] Hall TA (1999) BioEdit: a user-friendly biological sequence alignment editor and analysis program for Windows 95/98/NT. Nucleic Acids Symposium Series 41: 95–98.

[35] Lee W-J, Conroy J, Howell WH, Kocher TD (1995) Structure and evolution of teleost mitochondrial control regions. Journal of Molecular Evolution 41: 54–66. 7608989 10.1007/BF00174041

[36] Countway PD, Gast RJ, Savai P, Caron DA (2005) Protistan diversity estimates based on 18S rDNA from seawater incubations in the western North Atlantic. Journal of Eukaryotic Microbiology 52: 95–106. 15817114 10.1111/j.1550-7408.2005.05202006.x

[37] Meusnier I, Singer GAC, Landry J-F, Hickey DA, Hebert PDN, et al (2008) A universal DNA mini-barcode for biodiversity analysis. BMC Genomics 9 214: 214. 10.1186/1471-2164-9-214 PMC2396642 18474098

[38] McGaw IJ, Reiber CL (2000) Integrated physiological responses to feeding in the blue crab *Callinectes sapidus* . The Journal of Experimental Biology 203: 359–368. 10607545 10.1242/jeb.203.2.359

[39] Reichert MJM, Van Der Veer HW (1991) Settlement, abundance, growth and mortality of juvenile flatfish in a subtropical tidal estuary (Georgia, U.S.A.). Netherlands Journal of Sea Research 27: 375–391.

[40] Witting DA, Able KW (1993) Effects of body size on probability of predation for juvenile summer and winter flounder based on laboratory experiments. Fishery Bulletin 91: 577–581.

[41] Sheppard SK, Bell J, Sunderland KD, Fenlon J, Skervin D, et al (2005) Detection of secondary predation by PCR analyses of the gut contents of invertebrate generalist predators. Molecular Ecology 14: 4461–4468. 16313606 10.1111/j.1365-294X.2005.02742.x

[42] Nuttal MA (2010) Historical recount of the Great South Bay ecosystem, Long Island, New York, and a quantitative assessment of the ecosystem structure of Great South Bay using Ecopath. Stony BrookNY: Stony Brook University. 212 p.

