# Supplementary material for: A New PCR-Based Method Shows That Blue Crabs (Callinectes sapidus (Rathbun)) Consume Winter Flounder (Pseudopleuronectes americanus (Walbaum))
Source: PLoS One. 2014 Jan 13;9(1):e85101. doi: 10.1371/journal.pone.0085101 (PMC3890304; doi:10.1371/journal.pone.0085101)
Supplement: Figure S1 — Pseudopleuronectes americanus mitochondrial D-loop sequences. The alignment starts from position 1 of GenBank Accession Number U12068, and compares U12068 to sequences recovered with the WF208 primer pair from wild blue crab gut contents spiked with winter flounder tissue (HB-WF208), from feeding experiment crabs (GenBank Accession KF183646, FC2-WF208, FC4-WF208), and from wild crabs (WC14-WF208, WC15-WF208, WC40-WF208, and WC46-WF208). The locations and sequences of primers described in Table 1 are also shown. (PDF) [file pone.0085101.s001.pdf]

Figure S1. ***Pseudopleuronectes americanus*** **mitochondrial D-loop sequences.** The alignment starts from position 1 of GenBank Accession Number U12068, and compares U12068 to sequences recovered with the WF208 primer pair from wild blue crab gut contents spiked with winter flounder tissue (HB-WF208), from feeding experiment crabs (GenBank Accession KF183646, FC2-WF208, FC4-WF208), and from wild crabs (WC14-WF208, WC15-WF208, WC40-WF208, and WC46-WF208). The locations and sequences of primers described in Table 1 are also shown.

```
                     10         20         30         40         50         60         70         80         90        100        110        120
primers                                                                              WF200f 5'ATAATG AACTAGGACA TCT
U12068       CTTATAATGC ATGCGCTGAA AGTTTTCAAG TACATGTATG TAATAACACC ATATATTTAT AGTAACCATT TTATATAATG AACTAGGACA TCTATGTATA ATAACCTAAT CTAGTATTAT
KF183646     CTTATAATGC ATGCGCTGAA AGTTTTCAAG TACATGTATG TAATAACACC ATATATTTAT AGTAACCATT TTATATAATG AACCAGGACA TCTATGTATA ATAACCTAAT CTAGTATTAT
FC2-WF208                                                                                                                             CTAGTATTAT
FC4-WF208                                                                                                                        TAAT CTAGTATTAT

                    130        140        150        160        170        180        190        200        210        220        230        240
primers                                  WF270r 3'G ATTTACTGAA ACCTATT          WF310r 3'CATCTG AAAGTCCAGG AC
U12068       AGCACTCATT CATCAACATT TCTAACTAAG ATTTACTGAA ACCTATTTTT ACACTAATCT TACACATCTG AAAGTCCAGG ACCAGTCGAA ATTTAAGACC GAACACAACA CTCATCGGTC
KF183646     AACACTCATT CATCAACATT TTTAACTAAG ATATACTAGA ACCTATTTTA ACACTAATAT TACACATTTG AAAGTCCAGG ACCAGTCGAA ATTTAAGACC GAACACAACA CTCATCGGTC
HB-WF208       CACTCATT CATCAACATT TCTAACTAAG ATTTACTAAA ACCTATTTTT ACACTAACCT TACACATCTA AAAGTCCAGG ACCAGTCGAG ATTTAAGACC GAACACAACA CTCATCAGTC
FC2-WF208    AACACTCATT CATCAACATT TTTAACTAAG ATCTACTAGA ACCTATTTTA ACACTAATAT TACACATCTG AAAGTCCAGG ACCAGTCGAA ATTTAAGACC GAACACAACA CTCATCGGTC
FC4-WF208    AACACTCATT CATCAACATT TTTAACTAAG ATATACTAGA ACCTATTTTA ACACTAATAT TACACATTTG AAAGTCCAGG ACCAGTCGAA ATTTAAGACC GAACACAACA CTCATCGGTC
WC14-WF208         CATT CATCAACATT TCTAACTAAG ATCTACTARA ACCTATTTTW ACACTAAYAT TACACATCTR AAAGTCCAGG ACCAGTCGAR ATTTAAGACC GAACACAACA CTCATCRGTC
WC15-WF208     CACTCATT CATCAACATT TCTAACTAAG ATCTACTARA ACCTATTTTW ACACTAATAT TACACATCTR AAAGTCCAGG ACCAGTCGAR ATTTAAGACC GAACACAACA CTCATCRGTC
WC40-WF208       CTCATT CATCAACATT TCTAACTAAG ATCTACTARA ACCTATTTTW ACACTAACAT TACANATCTR AAAGTCCAGG ACCAGTCGAR ATTTAAGACC GAACACAACA CTCATCRGTC
WC46-WF208              CATCAACATT TCTAACTAAG ATCTACTARA ACCTATTTTW ACACTAACAT TACACATCTR AAAGTCCAGG ACCAGTCGAR ATTTAAGACC GAACACAACA CTCATCRGTC

                    250        260        270        280        290        300        310        320        330        340        350        360
primers                            WF400r 3'TCCAA CTCAAATTCG TAT
U12068       GAGTTATACC AAGACTCAAA ATCTCTCCAA CTCAAATTCG TATGTAGTAA GAGCCTACCA ACCGGTGATT CCTTAATGAT AACGGTTATT GAAGGTGAGG GACAAAAATT GTGGGGGTTT
KF183646     GAGTTATACC AAGACTCAAA ATCTCTCCAA CTCAAATTTG TATGTAGTAA GAGCCTACCA ACCGGTGATT TCTAAATGAT AACGGTTATT GAAGGTGAGG GACAAAAACT GTGGGGGTTT
HB-WF208     GAGTTATACC AAGACTCAAA ATCTCTCCAA CTCAAATTCG TAT
FC2-WF208    GAGTTATACC AAGACTCAaA ATCTCTCCAA CTCAAATTCG TAT
FC4-WF208    GAGTTATACC AAGACTCAAA ATCTCTCCAA CTCAAATTCG TAT
WC14-WF208   GAGTTATACC AAGACTCAAA ATCTCTCCAA CTCAAATTCG TAT
WC15-WF208   GAGTTATACC AAGACTCAAA ATCTCTCCAA CTCAAATTCG TAT
WC40-WF208   GAGTTATACC AAGACTCAAA ATCTCTCCAA CTCAAATTCG TAT
WC46-WF208   GAGTTATACC AAGACTCAAA ATCTCTCCAA CTCAAATTCG TAT

                    370        380        390        400        410        420        430        440        450        460        470        480
primers                      FF_2 3'catttggtt cctacttcag g                                                              FF_3 3'catacg actcgttacc ca
U12068       CACTCGGTGA ACTATTCCTG GCATATGGTT CCTACTTCAG GGCCATAAAT TGATATTATT CCCCACACTT TCATCGACGC TTACATAAGT TAATGTTGAT AATACATACG ACTCGTTACC CAA
KF183646     CACTCGGTGA ACTATTCCTG GCATTTGGTT CCTACTTCAG GGCCATAAAT TGATATTATT CCCCACACTT TCATCGACGC TTACATAAGT TAATGTTGAT AATACATACG ACTCGTTACC CAA
```
