# Supplementary material for: A New PCR-Based Method Shows That Blue Crabs (Callinectes sapidus (Rathbun)) Consume Winter Flounder (Pseudopleuronectes americanus (Walbaum))
Source: PLoS One. 2014 Jan 13;9(1):e85101. doi: 10.1371/journal.pone.0085101 (PMC3890304; doi:10.1371/journal.pone.0085101)
Supplement: Figure S2 — Calinectes sapidus coxI sequences. The alignment starts from position 1276 of GenBank Accession Number NC_006281 and compares NC_006281 to the consensus of sequence recovered from wild blue crabs by the Unibar primer pair. The locations and sequences of primers described in Table 1 are also shown. (PDF) [file pone.0085101.s002.pdf]

**Figure S2.** ***Calinectes sapidus coxI* sequences.** The alignment starts from position 1276 of GenBank Accession Number NC_006281 and compares NC_006281 to the consensus of sequence recovered from wild blue crabs by the Unibar primer pair. The locations and sequences of primers described in Table 1 are also shown.

```
                      10         20         30         40         50         60         70         80         90        100
primers       ~~~Uni-MinibarF1 5'TC CACTAATCAC AARGATATTG GTAC
NC_006281     ATGCAACGAT GATTCTTTTC TACAAATCAT AAAGACATTG GTACATTATA TTTCATTTTT GGAGCATGAT CTGGGATAGT AGGTACATCA CTTAGTTTAA
Unibar        ~~~~~~~~~~ ~~~~~~~~~~ ~~~~~~~~~~ ~~~~~~~~~~ ~~~~~~~~~~ ~~~~-TTTTT GGAGCATGCT CTGGGATAGT AGGTACATCA CTTAGTTTAA

                     110        120        130        140        150        160        170        180        190
primers                                                                                                                                                                                  Uni-MinibarR1 3'GCTCATGCC TTCATTATGA TTTTC
NC_006281     TCATTCGAGC TGAACTAGGA CAACCTGGAA CCCTTATTGG AAACGACCAA ATTTATAACG TTGTAGTCAC AGCTCACGCC TTTGTTATAA ttttc
Unibar        TCATTCGAGC TGAACTAGGA CAACCTGGAA CCCTTATTGG AAACGACCAA ATTTATAACG TTGTAGTCAC AGCTCATGCC TTCATTATGA TTTTC
```
