# Supplementary material for: A New PCR-Based Method Shows That Blue Crabs (Callinectes sapidus (Rathbun)) Consume Winter Flounder (Pseudopleuronectes americanus (Walbaum))
Source: PLoS One. 2014 Jan 13;9(1):e85101. doi: 10.1371/journal.pone.0085101 (PMC3890304; doi:10.1371/journal.pone.0085101)
Supplement: Table S1 — Number of sequence differences (mismatches plus insertion/deletions) between mitochondrial control region primers listed in Table 1 and control region sequences from flatfish and blue crab. (PDF) [file pone.0085101.s003.pdf]

Table S1. Number of sequence differences (mismatches plus insertion/deletions) between mitochondrial control region primers listed in Table 1 and control region sequences from flatfish and blue crab.

Species[a]	WF200f	WF270r	WF310r	WF400r	FF_A	FF_2	FF_3
**Family Pleuronectidae**							
Pseudopleuronectes americanus	0	0	0	0	nd	1	0
Limanda ferruginea**	2	6	5	8	nd	1	2
Glyptocephalus cynoglossus**	5	7	5	8	nd	nd	nd
Hippoglossoides platessoides**	4	8	4	5	nd	0	0
Hippoglossus hippoglossus**	4 - 5[c]	6 - 7[c]	2	6 - 7[c]	0	0	0
Reinhardtius hippoglossoides	4	8	3	10	0	0	0
Pseudopleuronectes herzensteini	1	4 - 6[c]	4 - 5[c]	3	0	1	nd
Platichthys stellatus	2	6	9	6	0	0	0
Platichthys flesus	3	7	9	5	nd[b]	0	0
Kareius bicoloratus	1	6	8 - 9[c]	4	0	nd	nd
Lepidopsetta mochigarei	2	6	4	5	1	0	nd
Pleuronectes platessa	2	6	8	4	nd	0	0
Acanthopsetta nadeshnyi	3	5	4	6	1	0	nd
Cleisthenes pinetorum	5	6	7	5	0/11[d]	0	nd
Glyptocephalus stelleri	6	7	5	9	nd	0	nd
Hippoglossoides dubius	4	5	5	7	1	0	nd
Hippoglossus stenolepis	4	7	4	7	0	0	0
Microstomus achne	4 - 6[c]	4	3 - 4[c]	7	0	1/15[d]	nd
Verasper moseri	7	6	3	5	0 - 2[c]	0	0
Verasper variegatus	5 - 7[c]	6 - 7[c]	4 - 6[c]	7 - 8[c]	0	0	0
**Family Paralichthyidae**							
Paralichthys dentatus**	4/14[d]	6	10	8	nd	nd	nd
Paralichthys olivaceus	9	7	10	8	0	0	0
Etropus microstomus**	nd	nd	nd	nd	nd	nd	nd
Hippoglossina oblonga**	nd	nd	nd	nd	nd	nd	nd
**Family Scophthalmidae**							
Scophthalmus aquosus**	nd	nd	nd	nd	nd	nd	nd
**Family Portunidae**							
Callinectes sapidus	na[e]	na[e]	na[e]	na[e]	6	na[e]	na[e]

nd: no sequence available for the primer binding site.
** indicates species with distributions overlapping winter flounder.
[a]Common name and distribution (data from www.fishbase.us accessed July 21, 2012), plus GenBank accession numbers for flatfish:

Pseudopleuronectes americanus (Walbaum, 1792), winter flounder, northwest Atlantic; U12068.1
Limanda ferruginea (Storer, 1839), yellowtail flounder, northwest Atlantic**; U12064.1
Glyptocephalus cynoglossus (Linnaeus, 1758), witch flounder, north Atlantic**; U12070.1
Hippoglossoides platessoides (Fabricius, 1780), American plaice, north Atlantic**; U12059.1
Hippoglossus hippoglossus (Linnaeus, 1758), Atlantic halibut, north Atlantic**; AM749122.1, AM749123.1, AM749124.1, AM749125.1
Reinhardtius hippoglossoides (Walbaum, 1792), Greenland halibut, circumglobal (northern hemisphere); AM749130.1
Pseudopleuronectes herzensteini (Jordan & Snyder, 1901), yellow striped flounder, northwest Pacific; AB447559.1, AY671932.1, AY671933.1, AY671934.1
Platichthys stellatus (Pallas, 1787), starry flounder, north Pacific; EF424428.1
Platichthys flesus (Linnaeus, 1758), European flounder, northeast Atlantic; EU075178.1
Kareius (*Platichthys*) *bicoloratus* (Basilewsky, 1855), stone flounder, northwest Pacific; AY67135.1, AY671936.1, AY671937.a, AP002951.1
Lepidopsetta mochigarei (Snyder, 1911), dusky sole, northwest Pacific; AB238948
Pleuronectes platessa (Linnaeus, 1758), European plaice, northeast Atlantic; EU075179.1
Acanthopsetta nadeshnyi (Schmidt, 1904), scale-eye plaice, north Pacific, AB238947.1
Cleisthenes pinetorum (Jordan & Starks, 1904), sôhachi, northwest Pacific, AB238949.1
Glyptocephalus stelleri (Schmidt, 1904), blackfin flounder, northwest Pacific, AB238944.1
Hippoglossoides dubius (Schmidt, 1904), flathead flounder, northwest Pacific, AB238496.1
Hippoglossus stenolepis (Schmidt, 1904), Pacific halibut, north Pacific; AM749128.1, AM749129.1
Microstomus achne (Jordan & Starks, 1904), slime flounder, northwest Pacific; AF104861.1, AY671924.1, AY671925.1, AY671926.1, AY671927.1
Verasper moseri (Jordan & Gilbert, 1898), Barfin flounder, northwest Pacific; AB207249.1, EF025506.1
Verasper variegatus (Temminck & Schlegel, 1846), spotted halibut, northwest Pacific; AB218654.1, AB522914.1 to AB522933.1
Paralichthys dentatus (Linnaeus, 1766), summer flounder, northwest Atlantic**; AF022712.1, AF082187.1
Paralichthys olivaceus (Temminck & Schlegel, 1846), bastard halibut, western Pacific; NC_002386.1
Etropus microstomus (Gill, 1864), smallmouth flounder, northwest Atlantic**; no control region sequence available
Hippoglossina oblonga (Mitchill, 1815), American fourspot flounder, northwest Atlantic**; no control region sequence available
Scophthalmus aquosus (Mitchill, 1815), windowpane flounder, northwest Atlantic**; no control region sequence available

[b]Likely sequencing errors in this region
[c]Range given where the available sequences differ
[d]Available sequence only covers part of primer binding site (number of comparable bases given as denominator)
[e]Sequence could not be aligned with sufficient confidence (na, not applicable)
