# Supplementary material for: A New PCR-Based Method Shows That Blue Crabs (Callinectes sapidus (Rathbun)) Consume Winter Flounder (Pseudopleuronectes americanus (Walbaum))
Source: PLoS One. 2014 Jan 13;9(1):e85101. doi: 10.1371/journal.pone.0085101 (PMC3890304; doi:10.1371/journal.pone.0085101)
Supplement: Table S2 — Characteristics and amplification results of the 55 crabs from Shinnecock Bay used in wild crab analysis. (PDF) [file pone.0085101.s004.pdf]

Table S2. Characteristics and amplification results of the 55 crabs from Shinnecock Bay used in wild crab analysis.

Crab Number	Unibar	WF208	Sex	Carapace Width (mm)	Location	Date Caught	Volume of Gut Contents (µL)	Description of Gut Contents
WC1	Y	N	M	81.5	Site 1	9/13/2011	200	Liquid
WC2	Y	N	F	111.8	Site 1	9/13/2011	50	Sandy mud, liquid
WC3	Y	N	F	91.2	Site 1	9/13/2011	500	Sandy mud, liquid
WC4	N	N	F	74.7	Site 1	9/13/2011	100	Hard bits/shell
WC5	Y	N	F	76.8	Site 1	8/4/2011	100	Mostly light liquid
WC6	nd	nd	F	91.2	Site 1	8/4/2011	Empty	-
WC7	Y	N	M	105.0	Site 1	8/10/2011	1000	Lots of shell
WC8	Y	N	F	75.3	Site 1	8/10/2011	100	Mostly light liquid
WC9	Y*	N	M	82.1	Site 1	8/10/2011	200	Likely mostly crab
WC10	N	N	F	98.8	Site 1	8/18/2011	1200	Red, shelly
WC11	N	N	M	112.7	Site 1	8/18/2011	1000	Shelly
WC12	N	N	M	93.6	Site 1	8/25/2011	100	Dark mud/liquid
WC13	Y	N	F	90.2	Site 1	8/25/2011	90	Some mud/shell
WC14	Y*	**Y***	F	84.4	Site 1	8/4/2011	1100	Mud/shell
WC15	Y*	**Y***	F	95.7	Site 1	8/4/2011	400	Red, blood-like
WC16	Y	**Y**	M	78.8	Site 1	8/4/2011	150	Mud/shell
WC17	Y	**Y**	F	91.3	Site 1	8/4/2011	600	Shelly
WC18	Y	**Y**	M	73.7	Site 1	8/4/2011	250	Almost all mud
WC19	N	N	F	64.4	Site 1	8/10/2011	200	Almost all mud
WC20	Y*	N	F	85.7	Site 1	9/22/2011	75	Almost all mud
WC21	Y	N	F	89.5	Site 1	9/22/2011	120	Light liquid
WC22	Y	N	F	110.2	Site 2	7/28/2011	100	Light liquid/ crab
WC23	Y*	N	F	86.5	Site 2	7/28/2011	100	Light liquid/ crab
WC24	Y	N	F	79.0	Site 2	7/28/2011	800	Shelly/gunky
WC25	Y	N	F	68.5	Site 2	7/28/2011	200	Dark mud/liquid
WC26	Y*	N	M	96.7	Site 2	8/4/2011	1000	Shelly
WC27	Y*	N	F	87.1	Site 2	8/4/2011	250	Mud/gunk
WC28	Y	N	M	75.3	Site 2	8/4/2011	75	Sand/gunk
WC29	N	N	F	85.6	Site 2	8/4/2011	350	Dark mud/liquid

WC30	Y	N	M	76.7	Site 2	8/4/2011	250	Shelly (large pieces)
WC31	Y	N	F	90.6	Site 2	8/4/2011	450	Shelly (small pieces)
WC32	nd	nd	M	87.0	Site 2	8/4/2011	Empty	-
WC33	Y	N	F	100.3	Site 2	8/4/2011	750	Reddish/shelly
WC34	Y	N	M	67.1	Site 2	8/4/2011	300	Muddy/crab
WC35	Y	N	M	78.7	Site 2	8/10/2011	30	Almost empty
WC36	Y*	N	M	83.4	Site 2	8/10/2011	75	Dark liquid
WC37	Y	N	M	105.1	Site 2	8/10/2011	600	Dark/shelly
WC38	nd	nd	F	76.8	Site 2	8/10/2011	Empty	-
WC39	nd	nd	F	63.3	Site 3	8/4/2011	Empty	-
WC40	Y*	**Y***	M	88.4	Site 3	8/10/2011	500	Shelly
WC41	Y	**Y**	M	72.8	Site 3	8/10/2011	200	Shelly
WC42	Y	**Y**	M	103.5	Site 3	8/10/2011	100	Large shell pieces
WC43	N	N	M	75.3	Site 3	8/10/2011	500	Red/shelly
WC44	Y	**Y**	M	92.1	Site 3	8/10/2011	1700	Shelly
WC45	Y	**Y**	F	112.2	Site 3	8/10/2011	500	Dark/shelly
WC46	N*	**Y***	F	140.6	Site 3	8/18/2011	300	Reddish/shelly
WC47	nd	nd	M	75.2	Site 3	8/4/2011	Empty	-
WC48	Y	**Y**	M	81.7	Site 3	8/30/2011	300	Dark/shelly
WC49	Y	N	M	139.2	Site 3	8/30/2011	2700	Reddish/shelly
WC50	Y	N	F	124.5	Site 3	8/30/2011	180	Light liquid
WC51	Y	N	M	81.0	Site 3	8/30/2011	400	Dark/shelly
WC52	Y*	N	F	74.2	Site 3	8/30/2011	400	Brown/red/shelly
WC53	Y*	N	M	54.3	Site 3	8/18/2011	50	Liquid/crab
WC54	N	N	F	74.9	Site 3	9/13/2011	50	Dark liquid
WC55	Y	N	F	84.4	Site 3	9/22/2011	90	Little dark tissue/crab

PCR results for Unibar and WF208 primer pairs indicated Y, for amplification of expected size product; N, for no amplification product; or nd, PCR not done for crabs with empty guts.

* indicates sequenced PCR product.

Site 1 Lat: 40°51.895', Long 072°29.507'; Site 2 Lat: 40°52.878', Long 072°28.373'; Site 3 Lat: 40°51.392', Long: 072°27.594'.
